# Impact of Extended Dosing Intervals and Ipsilateral Versus Contralateral Boosting on mRNA Vaccine Immunogenicity in Mice

**DOI:** 10.3390/vaccines13030263

**Published:** 2025-03-01

**Authors:** Bin Lu, Omkar Chaudhary, Balaji Banoth, Janhavi Nadkarni, Wei Zong, Emilie Mausser, Hillary Danz, Mona Motwani, Sophie Ruiz, Donghui Zhang, Gopinath Nageshwaran, Bachra Rokbi, William Warren, Frank DeRosa, Sudha Chivukula

**Affiliations:** 1 mRNA Center of Excellence, Sanofi, 200 West St., Waltham, MA 02451, USA; bin.lu@sanofi.com (B.L.); omkar.chaudhary@sanofi.com (O.C.); emilie.mausser@sanofi.com (E.M.); hillary.danz@sanofi.com (H.D.); mona.motwani@sanofi.com (M.M.); frank.derosa@sanofi.com (F.D.); 2 Former Employee of Sanofi, 200 West St., Waltham, MA 02451, USA; 3 Translational and Early Development Biostatistics, Sanofi, 200 West St., Waltham, MA 02451, USAdonghui.zhang@sanofi.com (D.Z.); 4 mRNA Center of Excellence, Sanofi, 1541 Avenue Marcel Mérieux, 69280 MarcyL’Etoile, France; sophie.ruiz@sanofi.com; 5 Global Antigen Design, Sanofi, 200 West St., Waltham, MA 02451, USA; gopi.nageshwaran@sanofi.com (G.N.); william.warren@sanofi.com (W.W.); 6 Global Antigen Design, Sanofi, 1541 Avenue Marcel Mérieux, 69280 Marcy L’Etoile, France; bachra.rokbi@sanofi.com

**Keywords:** mRNA-LNP, vaccines, influenza, ipsilateral, contralateral, prime–boost interval, immunogenicity

## Abstract

Background: Although mRNA vaccines have the potential to be developed and deployed rapidly to combat infectious diseases, the ideal method of administration and boosting schedule strategy for generating optimal immunogenicity is an area of active research. We compared the immune responses resulting from different schedules for prime–boost and boosting either ipsilaterally or contralaterally in relation to the initial vaccine dose. Methods: Influenza hemagglutinin (HA) was used as a model antigen for different vaccination regimens in mice using both mRNA lipid nanoparticles (mRNA-LNP) and AF03-adjuvanted recombinant protein (rHA-AF03) vaccines. Results: Increasing the prime–boost interval resulted in higher levels of serum anti-HA IgG and functional antibody hemagglutination inhibition (HAI) responses in mRNA-LNP-vaccinated animals, which correlated with an induction of germinal center (GC) B cells and follicular helper T (Tfh) cells in lymph nodes. In addition, longer prime–boost intervals resulted in higher levels of IL-2 and TNF-α producing CD4+ T cells two weeks after boosting. The number of Ig-secreting long-lived plasma cells increased with the length of prime–boost intervals. Contralateral boosting resulted in an increase in HAI titers and GC B cells compared to an ipsilateral boost. However, significantly higher numbers of GC B cells were induced in the draining lymph nodes following ipsilateral boosting than in the non-draining lymph nodes. Conclusions: Overall, our data provides insights into the immune mechanisms of action of mRNA-LNP to develop the optimal vaccine regimen for mRNA vaccine platforms.

## 1. Introduction

As seen during the severe acute respiratory syndrome coronavirus 2 (SARS-CoV-2) pandemic, mRNA vaccines can effectively prevent serious illness from coronavirus disease (COVID-19) [1,2]. mRNA vaccines can be developed quickly using a standardized production platform, are simpler to manufacture than conventional vaccines, and have proven to be safe [3,4]. As such, there is growing interest in using this technology to prevent a variety of infectious diseases, including influenza, respiratory syncytial virus (RSV), human metapneumovirus (hMPV), and others [5]. These vaccines are thought to primarily stimulate the immune system through various lipid components of the lipid nanoparticle (LNP) [6]. LNPs act as intrinsic adjuvants, stimulating production of a variety of chemokines and cytokines, including CXCL10 (IP-10), GM-CSF, IL-6, TNF-α, type 1 IFN, IFN-γ, and IL-1 cytokines, as recently reviewed by Lee et al. [7]. This leads to recruitment of monocytes, neutrophils, and dendritic cells (DCs) to the injection site, inducing robust T follicular helper (Tfh) cell responses and GC formation in lymph nodes (LNs). Together, the mRNA-expressed antigen(s) from the LNP vaccine formulation at the injection site and draining LNs in the antigen presenting cells activate local and systemic immune responses, and lead to long-lasting antibody production [8,9].

The initial mRNA vaccines against SARS-CoV-2 are administered in a multiple-dose series to establish durable immunity. The first dose primes the naïve immune system against viral spike surface antigens and subsequent boost doses are administered to enhance antibody-mediated immune responses. Most non-live attenuated vaccines require multiple doses, spread over weeks, months, or years to provide strong and long-lasting immunity [10]. Currently approved mRNA COVID-19 vaccines have different intervals between the prime and boost doses (21 or 28 days) [1,2]. However, multiple published studies have demonstrated a general advantage for extended prime–boost intervals beyond the standard 4-week schedule, with one study even showing a 3.5-fold increase in titers when boosted at a 12-week interval [11,12,13,14]. Interestingly, a study in mice also correlated longer prime–boost intervals with greater neutralizing antibody titers and more durable immune responses, with six to eight weeks between doses showing the highest levels of antigen-specific IgG titers [15]. In a recent randomized clinical trial, longer intervals between prime and boost vaccinations against HIV demonstrated improved immune responses in HIV-uninfected Thai volunteers [16]. Further investigations are needed to better understand the immune mechanisms behind the benefit of alternative prime–boost schedules with mRNA vaccines.

As with other intramuscularly delivered vaccines, the current mRNA COVID-19 vaccine has been authorized [17,18,19] for administration in the deltoid muscle of the upper arm, without specific mention of the choice of the arm used for the first or second vaccination during the primary vaccination schedule. Recent clinical data on whether administering a boosting dose of a mRNA COVID-19 vaccine on the same side of the arm (ipsilateral) or opposite sides (contralateral) affects immune activation and/or vaccine efficacy is limited, and evidence for benefits derived from the injection site used for prime–boost vaccinations are conflicting [20,21,22]. Earlier studies using other vaccine formats showed that sequential doses of rabies vaccines and of pneumococcal and *Haemophilus* influenza type b vaccines administered ipsilaterally or contralaterally resulted in contradictory immunological responses [23,24]. Hence, the optimal methodology of prime–boost vaccine administration is still under debate, highlighting the need to improve our understanding of the mechanisms underlying this facet of vaccine immunogenicity.

Here, we show a body of work designed to elucidate some of the immune mechanisms associated with extended vaccination schedules for both mRNA and protein-based vaccines. Mice were immunized with an mRNA-LNP-encoding influenza HA or an adjuvanted recombinant HA protein from the A/Tasmania/503/2020 (H3N2) strain at 14-, 21-, 35-, 42-, and 56-day prime–boost intervals to compare differing effects of altering vaccination schedules. Serum antibody responses and immune cell activation in the spleen, draining and non-draining LNs, and bone marrow indicated benefits of extended boosting schedules. Additionally, an evaluation of the potential benefits of contralateral vs. ipsilateral administration was conducted, which clearly indicated that contralateral administration was superior to ipsilateral administration.

## 2. Methods

### 2.1. Preparation of mRNA-LNP and Characterization

mRNA transcripts encoding for the hemagglutinin protein of A/Tasmania/503/2020 (TAS-20 HA) were synthesized by in vitro transcription using RNA polymerase and a plasmid DNA template encoding the gene using N1-methyl pseudouridine (1MpU)-modified nucleotides. The mRNA precursor product was enzymatically capped (5′ Cap 1) and tailed (3′ polyA) and the resulting mRNA drug substance was analyzed for purity, integrity, and capping efficiency before storage (−20 °C).

The mRNA-LNP formulation was prepared as previously described [25], by mixing appropriate solutions of mRNA and a lipid mixture. Briefly, an ethanolic solution of a mixture of lipids (ionizable lipid, phosphatidylethanolamine, cholesterol, and polyethylene glycol-lipid) and an aqueous buffered solution of target mRNA-LNPs were combined at a fixed lipid and mRNA-LNP ratio under controlled conditions to yield a suspension of uniform LNPs. After ultrafiltration and diafiltration into a suitable diluent system, the resulting nanoparticle suspensions were diluted to a final mRNA concentration (1 mg/mL), filtered, and stored at −80 °C until use.

### 2.2. Preparation of Recombinant HA Protein Vaccines with Adjuvant

Recombinant hemagglutinin (rHA) from influenza A/Tasmania/503/2020 was manufactured and purified using a baculovirus expression vector system as described previously [26]. The squalene-based adjuvant AF03 was generated as previously described [27].

### 2.3. Immunization in Mice and Sample Collections

**Figure 1:**
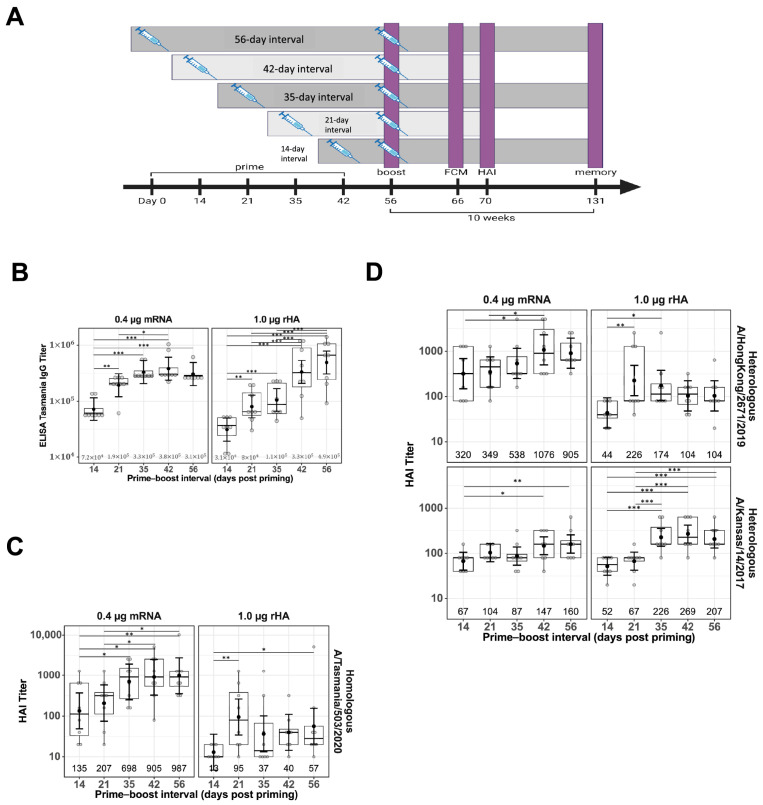
Impact of prime–boost interval on serum antibody titers. Serum samples were collected two weeks after prime–boost immunization with mRNA-LNP and rHA-AF03 vaccines. Samples were analyzed by IgG ELISA and hemagglutination inhibition (HAI) assays using homologous and heterologous influenza A viruses. (**A**) Schematic representation of the study design, showing different prime–boost intervals, sample collections, and assays. Created in BioRender. Mausser, E. (2025) https://BioRender.com/l56x824 (accessed on 14 February 2025). (**B**) Anti-HA IgG levels were analyzed by ELISA. (**C**) HAI titers using homologous virus A/Tasmania/503/2020 (H3N2) and (**D**) two heterologous viruses, A/Hong Kong/2671/2019 (H3N2) and A/Kansas/14/2017 (H3N2). Data are presented as boxplots of individual titers with geometric mean titers and 95% confidence intervals (black bars). * *p* < 0.05, ** *p* < 0.01, *** *p* < 0.001.

Female BALB/c mice (*Mus musculus*), 8–16 per treatment group, were immunized intramuscularly (IM) in the quadriceps (thigh muscle). Vaccination experiments were conducted using naïve mice. The priming dose was administered on Day 0, and the boosting dose was administered either contralaterally at Days 14, 21, 35, 42, or 56 or ipsilaterally at Day 21. A schematic representation of the study design, showing different prime–boost intervals, sample collections, and assays performed is shown in Figure 1A. Both the priming and boosting doses contained 0.4 μg mRNA in the LNP formulation or 1.0 μg rHA mixed with AF03, respectively. Terminal bleeds were performed via cardiac puncture under anesthesia. The spleen, draining LN (dLN), and bone marrow (femur) for the selected groups were collected upon study termination for flow cytometry analysis.

Spleen and dLNs were removed and placed in chilled RPMI with 10% FBS, 1% penicillin/streptomycin, and 1% L-glutamine (cRPMI) and kept on ice. Tissues were mechanically disrupted and the recovered cells were spun at 1200 rpm for 10 min. Spleen samples were treated with ammonium-chloride–potassium (ACK) lysis buffer to lyse red blood cells. Spleen and dLN cells were resuspended in cRPMI prior to cell counting, staining, and flow cytometry analysis.

To obtain bone marrow cells, femurs were recovered, and femur heads were cut off. A 25G needle was inserted into the femur and bone marrow was flushed into a 50 mL conical tube using ice-cold Hank’s balanced salt solution. Bone marrow was treated with ACK lysis buffer and washed several times with cDMEM before being cryopreserved using CTL-Cryo ABC (C.T.L., cat#: CTLC-ABC-500) in liquid nitrogen until further analysis.

### 2.4. Hemagglutination Inhibition (HAI) Assay

HAI assays were performed using A/Tasmania/503/2020 virus stocks, as previously described [25]. Briefly, the sera were treated with receptor-destroying enzyme at a dilution of 1:4 and incubated overnight at 37 °C. The enzyme was inactivated by a 30 min incubation at 56 °C, followed by the addition of six parts of PBS for a final dilution of 1/10. HAI assays were performed in V-bottom 96-well plates using four hemagglutinating units (4 HAU/25 μL) of virus and 0.5% turkey red blood cells. PBS or naïve serum were used as negative controls. The HAI titer was determined as the highest dilution of serum resulting in complete inhibition of hemagglutination.

### 2.5. Antibody IgG ELISA

Antibody IgG ELISA was performed using HA A/Tasmania/503/2020 protein captured on 96-well high-binding polystyrene plates (Nunc MaxiSorp Nunc-439454, Carlsbad, CA, USA) at a concentration of 1 µg/mL in carbonate–bicarbonate buffer. The plates were coated with 50 µL/well of HA in solution and incubated overnight (16 ± 4 h) at 2–8 °C, then washed 2–3 times with washing buffer (PBS 0.5% Tween 20) and blocked with blocking solution (1.0% BSA in PBS). Test samples, naïve control, and reference samples were diluted 1:50, followed by a 1:5 dilution in sample diluent (PBS 10% BSA 0.5% Tween 20) and added to wells in duplicate, followed by incubation at room temperature for 120 min. Anti-mouse horseradish peroxidase (HRP) IgG (Abcam 205719, Waltham, MA, USA) was added at a dilution of 1:20,000, followed by incubation at room temperature for 60 min. The plates were washed five times with the washing buffer. Excess HRP IgG was washed with PBS. SureBlue substrate (SERA CARE 5120-0075, Milford, MA, USA) was added to each plate and the reaction was stopped after 10 min of incubation away from light with hydrochloric acid (HCl). Plates were read at 450 nm using a Biotek Powerwave HT plate reader (BioTek Instruments, Inc.) Winooski, VT, USA. The anti-HA-specific antibody titers were expressed as the reciprocal of the highest serum dilution with an absorbance value > 0.3.

### 2.6. Flow Cytometry Analysis

The cellular immune responses of Tfh, GC, and B, in LN and spleen as well as memory B cells (MBCs) and long-lived plasma cells (LLPCs) from bone marrow were determined using Cytek Aurora 3L (V-B-R) flow cytometer with the SpectroFlow software, version 3.0.1. Intracellular cytokines produced by T cells were also analyzed. The methods for cellular and cytokine analyses are described below.

### 2.7. GC B and Tfh Cell Staining

To determine the frequency of GC in spleen or LN, the cells were first stained with LIVE/DEAD Near-IR (Invitrogen, Waltham, MA, USA; cat#: L34994) in PBS, followed by Fc-receptor blockade with anti-CD16/CD32 (BD Biosciences, Franklin Lakes, NJ, USA; cat#: 553142) in stain buffer (BD Biosciences, Franklin Lakes, NJ, USA; cat#: 554656) for 10 min at 4 °C. The cells were stained for 30 min on ice with the following fluorochrome-conjugated antibodies against surface markers panel in Brilliant Staining Buffer (BD Biosciences, Franklin Lakes, NJ, USA; cat#: 566349)): Tfh cells: [BD Biosciences, Franklin Lakes, NJ, USA] CD3-BV605 (cat#: 564009); [BioLegend, San Diego, CA, USA] CD4-BV421 (cat#: 100543), CXCR5-BV650 (cat#: 145517), PD1-PE (cat#: 109104GC B cells: [BD Biosciences, Franklin Lakes, NJ, USA] Fas-BV786 (cat#: 740906); [BioLegend] CD4/CD8/F4/80-PE/Dazzle 594 (cat#: 100566/100762/123146), CD19-BV711 (cat#: 115555), B220-PE/Cy5 (cat#: 103210), CD38-PE/Cy7 (cat#: 102718), IgD-Alexa Fluor 700 (cat#: 405730), CD138-BV605 (cat#: 142516), GL-7-FITC (cat#: 144604). Excess antibodies were washed away with staining buffer, and the cells were fixed with 2% paraformaldehyde for 15 min on ice, washed, and resuspended in staining buffer prior to flow cytometry analysis.

### 2.8. T Cells and Intracellular Cytokine Staining

To detect the antigen-specific T cell responses, 1 × 10^6^ splenocytes were stimulated with TAS-20 HA protein (2 µg/mL) (Protein Sciences, lot#: GKG113TS40) in cRPMI and incubated in U-bottom 96-well plates at 37 °C and 5% CO_2_. Golgi Plug and Golgi Stop (BD Biosciences, cat#: 555029; cat#: 554724) were added after two hours of stimulation, and proceeded overnight at 37 °C. DMSO served as a negative control with the background value from DMSO subtracted from all samples, and a combination of phorbol 12-myristate 13-acetate (20 ng/mL) and ionomycin (1 µg/mL) served as a positive control.

After 18 h of incubation at 37 °C, the cells were stained with LIVE/DEAD Near-IR in PBS, followed by Fc-receptor blockade with anti-CD16/CD32 in stain buffer for 10 min at 4 °C, and stained for 30 min on ice with the following fluorochrome-conjugated antibodies in Brilliant Stain Buffer: [BD Biosciences] CD3-BV605 (cat#: 564009), CD8-BV510 (cat#: 563068); [BioLegend] CD4-BV421 (cat#: 100543). Excess antibodies were washed away with stain buffer. Cells were fixed and permeabilized using the Cytofix/Cytoperm Buffer Set (BD Biosciences, cat#: 554714) and stained intracellularly with the following antibodies in 1× Perm/Wash Buffer: [BD Biosciences] TNF-α-FITC (cat#: 554418); [BioLegend] IL-2-PE (cat#: 503808), IFN-γ-PE-Cy7 (cat#: 505826), IL-4-Alexa Fluor 647 (cat#: 504110), IL-5-APC (cat#: 504306), IL-17A-PE/Dazzle 594 (cat#: 506938). After washing, the cells were resuspended in stain buffer.

### 2.9. Bone Marrow B Cell Staining

To determine the immunophenotype of MBCs and LLPCs, 1 × 10^6^ bone marrow cells were stained with LIVE/DEAD Near-IR in PBS, followed by Fc-receptor blockade with anti-CD16/CD32 in stain buffer for 10 min at 4 °C, and stained for 30 min on ice with the following fluorochrome-conjugated antibody panel in Brilliant Stain Buffer: MBCs: [BD Biosciences] CD3/CD14/CD56-BV650 (cat#: 564378/740486/748098), IgM-BV786 (cat#: 564028);[BioLegend] CD19-BV711, B220 PE-Cy5, CD38-PE-Cy7, Fas-BV605, CD138-BV605, IgD-Alexa Fluor 700, GL-7-FITC, CD24-APC (cat#: 138506), CD27-BV510 (cat#: 124229), IgG-PE/Dazzle 594 (cat#: 405330), IgA-biotin (cat#: 407004). Following a wash, cells were stained with streptavidin-PE (BioLegend. Cat#: 410504) secondary antibody in stain buffer for 30 min on ice.

LLPCs were surface-stained using the same antibody cocktail as described for the MBCs. In addition, fluorescent-labeled immunoglobulin (Ig) antibodies were replaced with clone-matched purified Ig antibodies: [BD Biosciences] IgM (cat#: 553405), IgG (cat#: 405301), IgA (cat#: 407002). Excess antibodies were washed away with stain buffer. Cells were fixed and permeabilized using the Cytofix/Cytoperm Buffer Set and stained intracellularly with IgG-PE/Dazzle 594, IgM-BV786, and IgA-biotin in 1× Perm/Wash Buffer. Following a wash, cells were stained with streptavidin-PE (BioLegend. Cat#: 410504) secondary antibody in stain buffer for 30 min on ice. Excess antibodies were washed away with staining buffer, and cells were fixed with 2% paraformaldehyde for 20 min on ice, washed, and resuspended in stain buffer. The absolute numbers of LLPCs (IgD−/DUMP−/CD138+/B220−) and MBC (IgD−/DUMP−/CD138−/B220+/CD19+/CD38+/GL7−) were analyzed.

### 2.10. Statistical Analyses

A linear model with treatment (mRNA-LNP and rHA-AF03 immunization), dosing interval, and their interaction was used to assess the interval effects on IgG ELISA, HAI, and flow cytometry data. Since GC B and Tfh responses from LNs were collected from both sides (left and right) for each animal, a linear mixed model was applied with factors (fixed effects) for treatment, boosting approaches, collection sides, and their interaction. An unconstructed correlation matrix was used to model the animal-specific errors. All dependent variables were log_10_-transformed prior to statistical analyses. Analyses were performed using R software 4.2.3 and GraphPad Prism 10 (version 10.0.0 for Windows, GraphPad Software, Boston, MA, USA).

## 3. Results

### 3.1. Increase in Functional Antibody Responses Generated with Increase in Boosting Intervals

To evaluate the impact of prime–boost interval on vaccine antibody responses, BALB/c mice were primed IM in the right thigh with either with mRNA-LNP or rHA-AF03 protein vaccine and homologous boosted with the mRNA-LNP or rHA-AF03 vaccine at 14- to 56-day intervals in the right (ipsilateral) or left (contralateral) thigh (Figure 1A). Serum was collected two weeks post-boosting and ELISA and HAI assays were performed to measure anti-HA IgG titers and functional antibody responses, respectively. Increasing prime–boost intervals generally increased post-boost IgG titers for both mRNA-LNP and rHA-AF03 vaccines (Figure 1B). The highest mean IgG titer was recorded for the mRNA-LNP vaccine at Day 42 prime–boost interval, whereas mean IgG titers for the rHA-AF03 vaccine increased steadily with increasing prime–boost intervals, with the highest mean titer reached at Day 56 prime–boost interval.

A similar trend was observed for HAI titers in the mRNA-LNP-vaccinated animals, where longer prime–boost intervals generally resulted in increasingly higher titers (Figure 1C). For the mRNA-LNP vaccine at a prime–boost interval of 14 days, the geometric mean neutralization titer (GMT) [95% CI] against the homologous A/Tasmania/503/2020 (H3N2) virus was 135 [49, 373] and reached 987 [356, 2735] for the 56-day prime–boost interval. In contrast, mice immunized with the rHA-AF03 vaccine showed dramatically different antibody response kinetics, with peak GMT titers of 95 [34, 264] and 226 [105, 488] at the 21-day prime–boost interval for the homologous A/Tasmania/503/2020 (H3N2). Additionally, although both mRNA-LNP and rHA-AF03 induced similar IgG titers, the mRNA-LNP vaccine induced a significantly higher ratio of functional antibodies to total IgG titers.

To better understand the differences in breadth of protection provided by these two vaccine modalities, sera samples from mice that received a prime–boost with homologous A/Tasmania/503/2020 (H3N2) antigen were tested for the ability to neutralize heterologous virus strains A/Hong Kong/2671/2019 (H3N2) and A/Kansas/14/2017 (H3N2) (Figure 1D). The general trend observed was similar to the homologous virus strain, where higher titers of functional antibodies were generated against the heterologous strains with increasing prime–boost intervals. Interestingly, the mRNA-LNP vaccine-induced better titers than the rHA-AF03 against A/Hong Kong/2671/2019 (H3N2) whereas the rHA-AF03 vaccine induced better titers than the mRNA-LNP A/Kansas/14/2017 (H3N2). These data suggest that different mechanisms of immune activation may play a role in these two different vaccine technology platforms.

### 3.2. Minimal Effect of Prime–Boost Interval on Production of Germinal Center B Cells (GC B) and Follicular Helper T (Tfh) Cells in Lymph Node and Spleen

**Figure 2:**
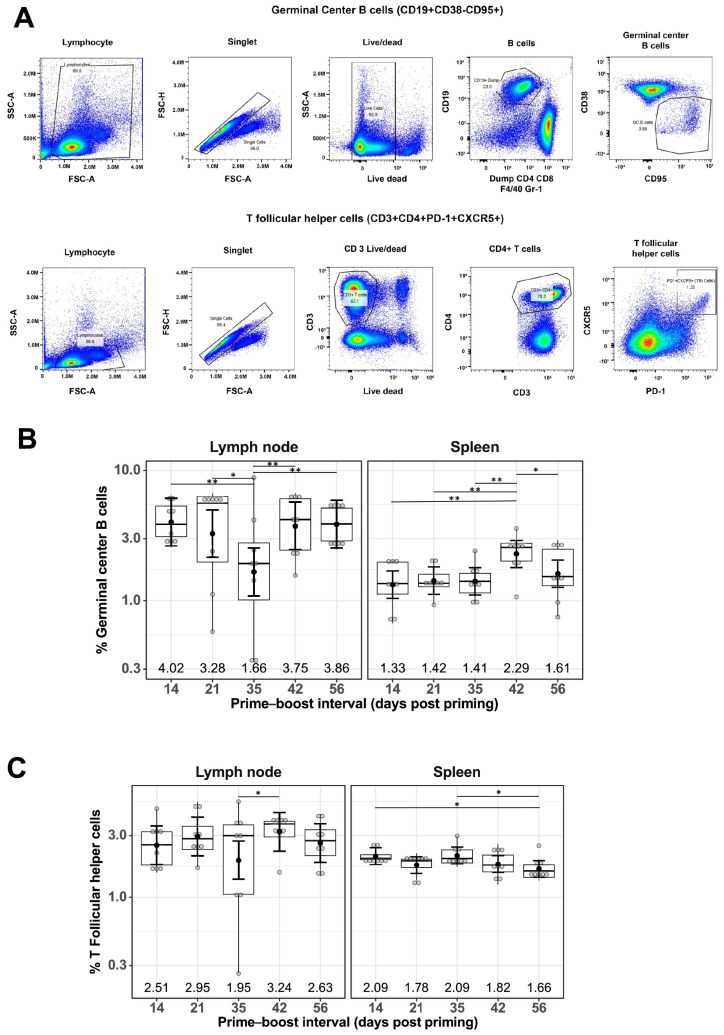
Effect of prime–boost interval on the generation of germinal center B cells and follicular helper T cells in the lymph nodes and spleen. (**A**) Representative flow cytometry plots showing gating strategy used to quantify GC B and Tfh cells derived from the lymph nodes and spleen of prime–boost vaccinated mice. Quantification of GC B cells (**B**) and Tfh cells (**C**) as described in the Materials and Methods. Data are presented as boxplots of the individual percentage of frequency with geometric mean frequencies and 95% confidence intervals (black bars). * *p* < 0.05, ** *p* < 0.01.

To understand the cellular responses following mRNA-LNP vaccination at different prime–boost intervals, flow cytometry staining of immune cells from the LNs and spleens was performed 10 days after boosting immunization. Specifically, GC B cells and Tfh cells, two cell populations essential for generating high-affinity antibodies, were investigated. The gating strategy for GC B cells included CD19+, CD38−, and CD95+, and those for Tfh cells were CD3+, CD4+, PD-1+, and CXCR5+. Representative flow cytometry plots showing the gating strategies used are shown in Figure 2A.

The percentage of GC B cells detected in LNs of mRNA-LNP-immunized mice remained generally constant at different prime–boost intervals, with a geometric mean frequency (GMF) of 3.16%, except for a slight decrease at the 35-day prime–boost interval, with a GMF of 1.66% [1.09%, 2.54%] (Figure 2B). A similar trend was observed for the percentage of GC B cells found in spleens for mRNA-LNP-immunized mice, with a GMF of 1.58% and the highest GMF of 2.29% [1.80%, 2.91%] at the 42-day prime–boost interval (Figure 2B). Similarly, the percentage of Tfh cells remained constant regardless of different prime–boost intervals, with a GMF of 2.62% in LNs and 1.88% in spleens of mRNA-LNP-immunized mice (Figure 2C).

### 3.3. Increased Production of CD4+ T Cells with Increase in Prime–Boost Interval

**Figure 3:**
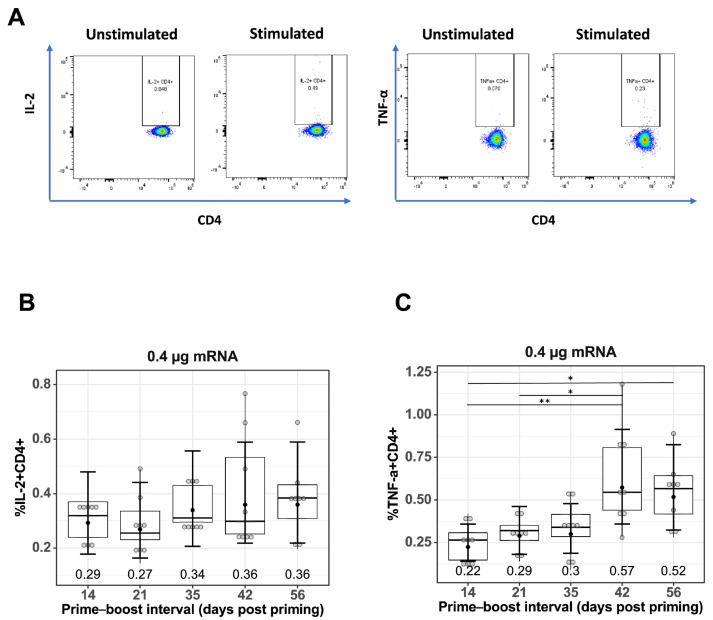
Effect of prime–boost interval on vaccine-induced CD4+ T cell responses. (**A**) Representative flow cytometry plots showing gating strategy used to quantify IL2+CD4+ and TNF-α+CD4+ cells derived from spleen of prime–boost vaccinated mice. Quantification of (**B**) IL-2+ CD4+ T cells and (**C**) TNF-α+CD4+ T cells in spleens of mice vaccinated with mRNA-LNP as described in the Materials and Methods. Data are presented as boxplots of the individual percentage of frequency with geometric mean frequencies and 95% confidence intervals (black bars). * *p* < 0.05, ** *p* < 0.01.

To measure antigen-specific T cell responses, spleen cells were stimulated with TAS-20 HA protein, the gating strategy for quantifying IL-2+CD4+ and TNF-α+CD4+ T cells is shown in Figure 3A. Quantification of IL-2+CD4+ (Figure 3B), and TNF-α+CD4+ (Figure 3C) T cells from prime–boost-immunized mice at different prime–boost intervals. Increasing the prime–boost interval from 14 days to 42 and 56 days resulted in higher mean levels of both IL-2+ and TNF-α+CD4+ T cells in the mRNA-LNP-immunized mice, with the GMF of IL-2+CD4+ T cells increasing from 0.29% (14 days) to 0.36% (42 days *p* = 0.564 and 56 days *p* = 0.564) and the GMF of TNF-α+CD4+ T cells increasing from 0.22% (14 days) to 0.57% (42 days, *p* = 0.006) and 0.52% (56 days, *p* = 0.014).

### 3.4. The Effect of Prime–Boost Intervals on the Induction of LLPCs and MBCs in Bone Marrow

**Figure 4:**
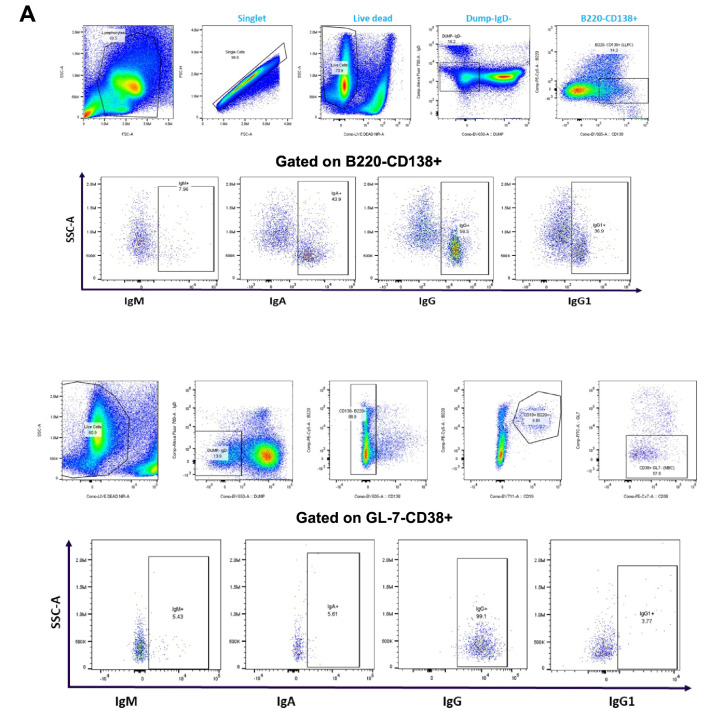

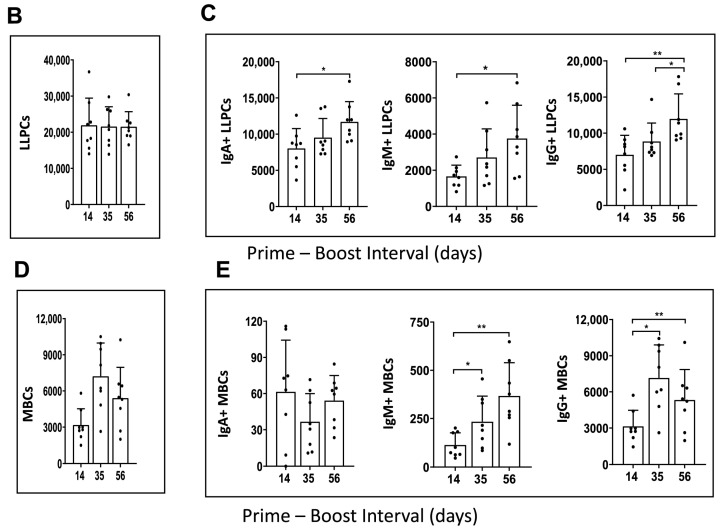
Immunophenotype analysis of vaccine-induced MBCs and LLPCs in bone marrow at different prime–boost intervals. (**A**) Representative flow cytometry plots showing gating strategy used to quantify LLPCs (upper panel) and MBCs (lower panel) derived from bone marrow cells that were harvested after 10 weeks post-boosting. (**B**) Quantification of absolute number of LLPCs. (**C**) LLPCs analyzed for different immunoglobulins. (**D**) Quantification of absolute number of MBCs. (**E**) MBCs analyzed for different immunoglobulins. Data are presented as bar charts showing the average of each cell population found in 1 × 10^6^ bone marrow cells and 95% confidence intervals (black bars). * *p* < 0.05, ** *p* < 0.01.

Durable, affinity-matured Ab responses associated with the induction of GC reactions give rise to LLPCs and MBCs [28]. Since we have reported that Tfh and GC B cells remain constant with varying prime–boost intervals, we analyzed bone marrow resident LLPCs and MBCs at 10 weeks after the second immunization. Representative flow plots showing the gating strategies used are shown in Figure 4A.

The results showed that mRNA-LNP vaccine induced a similar number of LLPCs with 14-, 35-, and 56-day prime–boost intervals (Figure 4B). However, the number of LLPCs producing immunoglobulins IgA, IgM, and IgG increased as the prime–boost interval increased from 14 to 35 and 56 days (Figure 4C). When the number of bone marrow resident MBCs was evaluated, an increase was observed from 14- to 35-day prime–boost time intervals and then declined when the interval increased to 56 days (Figure 4D). The numbers of IgA+, IgM+, and IgG+ MBCs varied with the different prime–boost intervals; for example, IgM+ MBCs increased with the prime–boosting interval from 14, to 35, to 56 days, while IgG+ MBCs increased between 14- and 35-day prime–boost intervals, then decreased with a 56-day prime–boost interval (Figure 4E). IgA+ MBCs were detected in lower numbers compared to IgM+ and IgG+ MBCs. The significance of these different kinetics needs to be investigated further. Taken together, these results suggest that the mRNA-LNP vaccine elicits stronger humoral immunity generated by MBC and LLPC responses as the prime–boost interval increases.

### 3.5. Minium Impact of Ipsilateral Versus Contralateral Vaccine Boosting on Functional Antibody Responses

**Figure 5:**
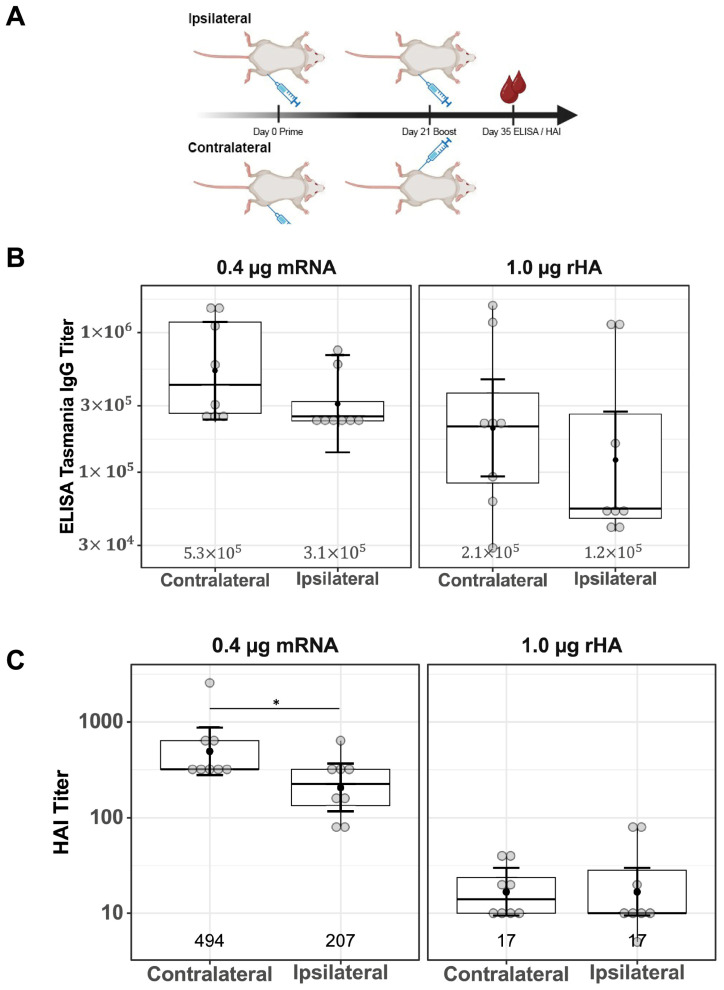
Effect of ipsilateral versus contralateral vaccine boosting on serum IgG titers and functional antibody responses. (**A**) Schematic of study design showing a 21-day prime–boost interval tested with one cohort of mice being boosted ipsilaterally and the other cohort boosted contralaterally; after 14 days (on Day 35), serum was collected and (**B**) ELISA and (**C**) HAI assays were performed. Created in BioRender. Mausser, E. (2025) https://BioRender.com/q66m571 (accessed on 14 February 2025). Data are presented as boxplots of individual titers with geometric mean titers and their 95% confidence intervals (black bars). * *p* < 0.05.

Differences in serum IgG titers and functional antibody responses comparing ipsilateral and contralateral vaccine boosting were investigated for both mRNA-LNP and rHA-AF03 vaccines. BALB/c mice were immunized (primed) IM in the right thigh, then boosted with mRNA-LNP or rHA-AF03 after 21 days either in the same right thigh (ipsilateral) or left thigh (contralateral). Serum was collected two weeks after boosting, and ELISA and HAI assays were performed to measure the anti-HA IgG titers and functional antibody responses, respectively (Figure 5A).

Contralateral versus ipsilateral boosting did not result in a significant difference in anti-HA IgG titers in both vaccine modalities (Figure 5B, mRNA-LNP *p* = 0.33, rHA-AF03 *p* = 0.35). Consistent with the previous results (Figure 1C), HAI titers were higher in the mRNA-LNP-immunized cohort than in the rHA-AF03 cohort. HAI titers were significantly higher in the contralaterally boosted mRNA-LNP animals, compared to the contralaterally boosted rHA-AF03 cohort (*p* < 0.001), the same significant increase was seen in the ipsilaterally boosted mRNA-LNP cohort compared to the ipsilaterally boosted rHA-AF03 cohort (*p* < 0.001) (Figure 5C). In contrast, there were no significant differences in functional serum antibody titers between the ipsilateral and contralateral boost in the rHA-AF03 cohorts (*p* = 1.0), while the contralateral mRNA-LNP cohort had marginally higher serum antibody titers than the ipsilateral mRNA-LNP (*p* = 0.04) cohort (Figure 5C). 

### 3.6. The Effect of Ipsilateral Versus Contralateral Vaccine Boosting on the Production of GC, Follicular Helper T Cells, and Vaccine-Induced CD4+ Cell Cytokines

**Figure 6:**
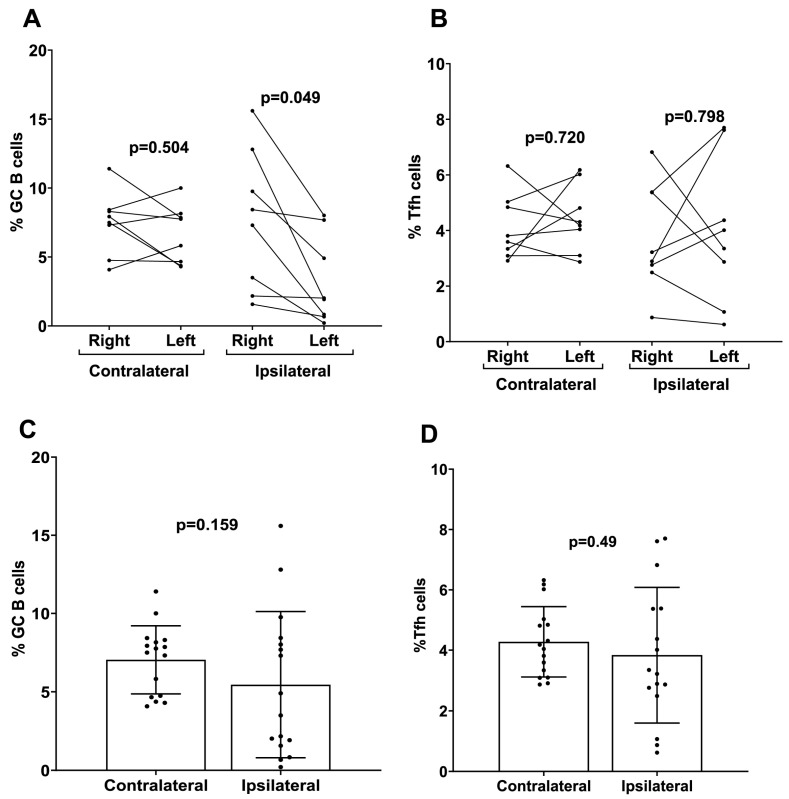
Analysis of germinal center B cells (GC B) and follicular helper T cells (Tfh) induced by contralateral and ipsilateral prime–boost mRNA-LNP vaccination. Quantification of (**A**) GC B and (**B**) Tfh cells derived from the LNs of ipsilateral and contralateral prime–boost-vaccinated mice. Cells were separately analyzed from the left side (non-draining) and right side (draining) LNs. Data are presented as dot plots with connecting dots representing data from the same animal: quantification of total (**C**) GC B and (**D**) Tfh cells from the LNs (both right and left sides pooled). Data are presented as bar charts showing the percentage of each cell population and 95% confidence intervals (black bars).

To investigate the mechanistic role of ipsilateral versus contralateral prime–boost immunization on mRNA-LNP vaccine efficacy, the number of GC B and Tfh cells were measured separately from draining and non-draining LNs in mice that received ipsilateral versus contralateral prime–boost vaccinations. There were higher numbers of GC B cells induced following ipsilateral boosting found in the draining LN (right side) for the mRNA-LNP-immunized mice compared to the number of GC B cells produced in the non-draining LN (left side) (Figure 6A). There was no significant difference in the Tfh cell numbers between draining and non-draining LNs regardless of ipsilateral versus contralateral boosting (mRNA-LNP ipsilateral *p* = 0.80 mRNA-LNP contralateral *p* = 0.72) (Figure 6B). When the total percentage of GC B and Tfh cells was analyzed in whole LNs (left + right), no significant differences were detected in the proportions of both cell populations in contralateral boosted mice compared to ipsilateral boosted mice for the mRNA-LNP cohort (Figure 6C,D).

## 4. Discussion

The widespread success of mRNA-LNP vaccination to prevent serious illness during the COVID-19 pandemic has undoubtedly shaped the future of mRNA-LNP technologies for influenza, RSV, SARS-CoV-2, and other respiratory viral vaccines [7,29,30]. The pharmacokinetics of different modalities, such as protein subunit, mRNA-LNP, and other forms of vaccines can have a dramatic impact on factors such as immunogenicity and reactogenicity through different effects on bio-distribution, antigen presentation, immuno-trafficking to lymphoid organs, etc. Understanding these mechanisms could lead to better mRNA-LNP vaccine designs and more efficient and effective vaccination strategies in the future. In the work presented here, we compared two vaccine modalities, mRNA-LNP and adjuvanted recombinant protein, with a focus on investigating two critical variables and their cellular mechanisms that can affect mRNA-LNP vaccine immunogenicity: the effect of prime–boost intervals and ipsilateral versus contralateral boosting.

To understand the mechanistic differences between the mRNA-LNP and protein subunit vaccines, we used a model influenza A (H3N2) HA antigen in both the mRNA-LNP vaccine and rHA-AF03 formulations. The first variable we investigated was the effect of increasing the length of the prime–boost interval on immune response. Previous studies with COVID-19 mRNA vaccines have shown that increasing the prime–boost interval can result in enhanced humoral and T cell responses [12,31,32]. Our results showed a similar increasing mean trend for the Influenza A mRNA-LNP vaccine with IgG titers, functional anti-HA antibody titers, and vaccine-induced T cells being highest at the longer prime–boost interval tested (42 or 56 days). Additionally, immunization with mRNA-LNP elicited higher functional antibody titers by HAI and improved the ratio of functional to total antibodies generated when compared to the rHA-AF03 protein vaccine (Figure 1). Clinical studies defining prime–boost intervals are recommended for future vaccine development involving mRNA-LNP vaccines. One such observational study by Hall et al. [31] evaluated humoral and cellular responses to two doses of BNT162b2 (COVID-19) mRNA-LNP vaccine indicated that delaying the boost up to 16 weeks before the second booster dose significantly enhanced anti-receptor-binding domain antibody titers as well as antigen-specific polyfunctional CD4+ and CD8+ T cells expressing IFN-γ and IL-2. A similar study [33] investigated the impact on responses to a third administration on participants who either had <89 days interval between the initial prime and boost with the BNT162b2 mRNA-LNP vaccine and those who had ≥89 days interval and found a similar advantage to increasing the boosting interval as stronger antibody responses were demonstrated.

To explore the cellular mechanisms underlying the observed serological responses, we examined the generation of GC B cells (CD19+CD38−CD95+) and Tfh cells (CD3+CD4+PD1+CXCR5+PD-1+) (Figure 2). Germinal centers play a critical role in vaccine-induced humoral immunity by mediating the production of protective antibodies and MBCs following mRNA-LNP vaccination [34]. Consistent with the increasing serum IgG and HAI titers, the percentage of splenic GC B cells also increased slightly with increasing prime–boost. Tfh cells play a fundamental role in the regulation of high-quality antibody responses. Robust induction of Tfh cells was observed post mRNA-vaccinations. In contrast to the GC B cells, and consistent with previous reports on mRNA-LNP vaccines [9,34,35], Tfh numbers remained at a steady level irrespective of the boosting interval [9,34,35].

T cell immunity is functionally heterogeneous; in addition to the CD4+ Tfh cells, which play key roles in the development of MBC, plasma cells, and antibodies, CD4+ Th1 cells support and enhance the quality of memory CD8+ T cell responses [36,37]. Previous clinical studies on prime and boost vaccination against SARS-CoV-2 infection showed that booster vaccination with mRNA-LNP improved SARS-CoV-2-specific T cell responses [38]. In our current study, increasing the time interval of boosting significantly increased Th1 CD4+ cells expressing TNF-α in mRNA-LNP-vaccinated animals (Figure 3C). There was a slight increase in Th1 CD4+ cells expressing IL-2 with increasing boost interval; however, this increase was not significant (Figure 3B).

B cell-mediated immunological memory consists of two major cellular components: long-lived, high-affinity antibody-secreting cells (LLPCs) that maintain continuous immunoglobulin secretion and MBCs, which are responsible for rapid recall responses [39]. The quality of the recall response is determined by the memory B cell repertoire, which undergoes affinity maturation and a class switch inside GC reactions [40]. Durable immune memory is known to be generated by mRNA-LNP vaccines where it was shown that numbers of functional MBCs increased from 3 to 6 months post vaccination [41,42,43]. To examine the effects of the prime–boost strategy on the generation of MBCs and LLPCs, we performed flow cytometric staining of bone marrow cells. The results showed that a prime–boost with mRNA-LNP vaccine induced LLPCs and MBCs 10 weeks after the second boost dose (Figure 4). However, the production of different immunoglobulins by LLPCs was consistently higher on Day 56 compared to Day 14 for the mRNA-LNP vaccine (Figure 4). Overall, our results demonstrated increased numbers of LLPCs and MBCs with longer boosting intervals, but due to experimental limitations, we cannot claim that these cells are antigen-specific. However, we believe that the overall increase in these immune effector cells is due to mRNA-LNP vaccination. To date, little is known about the effect of ipsilateral versus contralateral boosting on vaccine immunogenicity in humans, especially for mRNA vaccines. Additionally, conflicting results were demonstrated in the few existing studies that investigated these parameters. A recent observational study by Ziegler et al., comparing adults who received the second dose of COVID-19 BNT162b2 mRNA-LNP vaccine, demonstrated that secondary boosting induced a stronger immune response when choosing vaccine administration routes that allowed for drainage by the same LNs (i.e., ipsilateral) used for priming [22]. A retrospective cohort study in over 2 million subjects receiving the BNT162b2 vaccine showed that ipsilateral boosting probably resulted in better vaccine efficacy compared to contralateral boosting due to a robust local LN activation [21]. Our results demonstrated that although contralateral boosting resulted in slightly higher IgG and HAI titers in mice, there was no statistical significance when compared to ipsilateral boosting. We found a noticeable increase in the number of GC B cells induced at the draining LNs compared to the non-draining LNs following ipsilateral boosting of the mRNA-LNP vaccination, suggesting that mRNA-LNP vaccines are efficient in generating an immune response that is more dependent on the vaccination side selected for prime–boost immunization. While only homologous prime–boost strategies were investigated in this study, further investigation regarding heterologous prime–boosting would be beneficial for mRNA vaccination strategies.

A recent study in mice immunized with mRNA-LNP vaccines expressing SARS-CoV-2 Omicron antigens demonstrated comparable immune responses and equivalent protection against SARS-CoV-2 infection when vaccination was administered via either ipsilateral or contralateral boosting [44]. Previous reports demonstrated that GC formation and the induction of HA-specific Tfh cells occurred exclusively in vaccine-draining LNs in rhesus macaques vaccinated with mRNA-LNP vaccine encoding influenza H10/HA protein [35]. Another recent study in mice using the BNT162b2 vaccine also demonstrated an advantage of ipsilateral boosting over contralateral boosting, whereby the positive selection and plasma cell differentiation of pre-existing GC B cells and high-affinity RBD-specific antibodies were rapidly generated; this was demonstrated even in the case of ipsilateral boosting with a different antigen [45]. Both these studies showed similar results to those reported in our current study in relation to the higher numbers of GC B cells in draining versus non-draining LNs. A study in mice by Kuraoka et al. showed similar results to our study, they used recombinant HA protein vaccination and demonstrated that serum antibody responses and the overall production of GC B cells did not change due to ipsilateral versus contralateral boosting with homologous antigens [46]. We acknowledge the limitation that preclinical research utilizing inbred mouse strains may not reflect findings in genetically diverse human populations, as the narrow genetic background of these models may not accurately reflect the complex interplay of genetic factors observed in human disease.

## 5. Conclusions

In summary, this study demonstrated that longer prime–boost intervals in immunized mice resulted in higher levels of serum anti-HA IgG and functional antibody responses to novel mRNA-LNPs expressing the TAS-20 HA antigen. The induction of GC B cells and Tfh cells in the LNs was correlated with longer prime–boost intervals, in addition to the generation of higher levels of vaccine-induced T cells. The strategic timing of the prime–boost interval for mRNA-LNP vaccination may result in greater immunogenicity and stronger protection. The importance of choosing ipsilateral or contralateral prime–boosting for vaccination is often overlooked, yet there seems to be mounting evidence for improved immune induction in ipsilateral prime–boost regimens. Our results showed significantly higher numbers of GC B cells induced in the draining LN following ipsilateral boosting compared to the number of GC B cells produced in the non-draining LN. In general, these results emphasize the multiple factors that should be considered to enhance mRNA-LNP vaccination immunogenicity, and in the future, investigators can leverage these strategies to optimize vaccine regimens to decrease the disease burden of mRNA-LNP vaccine-preventable infectious diseases.

## Data Availability

The datasets generated during and/or analyzed during the current study are available from the corresponding author on reasonable request.
